# A Self-Guided Mobile Mindfulness Intervention Embedded in Daily Routines for Adults With Mild to Moderate Psychological Distress: Randomized Controlled Trial

**DOI:** 10.2196/98056

**Published:** 2026-07-15

**Authors:** Xichun Wu, Jackelyn De Alwis, Zenan Dou, Hoyin Lo, Sisi Wang, Jingyi Wu, Wei Xu

**Affiliations:** 1 Beijing Key Laboratory of Applied Experimental Psychology, National Demonstration Center for Experimental Psychology Education (Beijing Normal University), Faculty of Psychology, Beijing Normal University, 19 Xinjiekouwai Street, Haidian District, Beijing, 100875, China, +86 010 58806836; 2 Pause Lab, Beijing, China

**Keywords:** mindfulness, mobile intervention, digital mental health, anxiety, depression, randomized controlled trial

## Abstract

**Background:**

Mobile mindfulness interventions have shown promise for reducing anxiety and depressive symptoms, but sustaining engagement remains a persistent challenge. Many digital programs still rely on formal practice that requires dedicated time, which may be difficult to integrate into daily life.

**Objective:**

This randomized controlled trial evaluated Habitual Mindfulness Practice (HMP), a self-guided mobile mindfulness intervention that embeds brief practices into recurring daily routines, among adults with mild to moderate psychological distress. Outcomes were compared with those of Traditional Mindfulness (TM), Mindfulness-Based Psychoeducation (MBP), and a waitlist control (WL).

**Methods:**

Adults aged 18 to 65 years with mild to moderate symptoms of anxiety or depression were randomly assigned in a 1:1:1:1 ratio to HMP, TM, MBP, or WL (N=686). All procedures were conducted online, and the intervention was fully self-guided, with outcomes assessed using self-report measures. The intervention lasted 21 days, with assessments conducted at baseline, postintervention, and 3-month follow-up. Primary outcomes were depressive and anxiety symptoms. Secondary outcomes included mindfulness, cognitive emotion regulation, affective balance, and interpersonal difficulties. Postintervention group differences were examined, controlling for baseline scores, and longitudinal trajectories were evaluated across the active intervention conditions.

**Results:**

At postintervention, significant group effects were observed for depressive symptoms (*F*_3,681_=28.67, *P*<.001, ηp²=0.11) and anxiety symptoms (*F*_3,681_=30.11, *P*<.001, ηp²=0.12). Both HMP and TM showed lower depressive and anxiety symptom scores than MBP and WL. TM showed lower postintervention anxiety than HMP (*P*=.04), whereas depressive symptoms did not differ significantly between HMP and TM (*P*=.63). The mindfulness practice conditions also showed more favorable postintervention outcomes for mindfulness, affective balance, interpersonal difficulties, and emotion regulation. Improvements in depressive and anxiety symptoms were generally maintained at follow-up among the active intervention conditions, although maintenance of secondary outcomes varied across measures. Postintervention outcome data were available for 55.2% (379/686) of randomized participants, and follow-up outcome data were available for 24.1% (124/515) of participants in the active intervention conditions. HMP and TM did not differ significantly in practice duration, engagement, or satisfaction.

**Conclusions:**

A routine-embedded, self-guided mobile mindfulness intervention may be a feasible approach for reducing mild to moderate psychological distress. HMP produced benefits broadly comparable to those of traditional app-delivered mindfulness, but it did not confer advantages in engagement or short-term efficacy.

## Introduction

Psychological distress, particularly depression and anxiety, is associated with impairments in everyday functioning, including difficulties in emotion regulation, disrupted affective balance, and strain in interpersonal processes [1-3]. Addressing these challenges is, therefore, not only a matter of symptom reduction but also of improving broader adaptive functioning. Among the approaches developed for this purpose, mindfulness-based interventions (MBIs) have received increasing attention because they aim to cultivate present-moment awareness, nonjudgmental observation, and more flexible self-regulation [4]. Evidence indicates that mindfulness practice can reduce depressive and anxiety symptoms while also supporting emotion regulation, well-being, and interpersonal functioning [5-8].

However, the impact of MBIs in real-world settings depends not only on their clinical effectiveness but also on their accessibility. A substantial proportion of individuals experiencing psychological distress do not seek or receive formal treatment, contributing to a persistent treatment gap in mental health care [9]. In response, MBIs have increasingly been adapted for digital delivery to improve accessibility and scalability. Digital mindfulness interventions can reduce symptoms of depression and anxiety, even in relatively brief programs [10,11], and therefore represent a promising approach for reaching individuals with unmet mental health needs.

Despite these advantages, accessibility does not necessarily translate into sustained engagement. Digital mindfulness programs commonly provide psychoeducational content to introduce mindfulness principles before guiding users through experiential practice. This structure is consistent with MBIs, which typically include instruction in meditation practices as part of the intervention model [12]. This experiential component is central to the clinical logic of mindfulness, particularly for psychological distress, where rumination, avoidance, attentional difficulty, and emotional reactivity often unfold in daily life [11]. Through repeated practice, individuals may become more able to recognize thoughts and emotions as they arise and to meet them with greater awareness and less reactivity [13,14]. Yet many practice-based digital programs rely on structured sessions that require dedicated time and attention. Although such formats are clinically meaningful, they may be difficult to sustain in everyday life, particularly for individuals with busy or irregular routines [15]. For self-guided mobile interventions, the challenge is not only to deliver mindfulness content but also to help the practice become available within everyday routines.

One potential way to address this limitation is to embed mindfulness practice more explicitly within recurring daily routines. Habitual Mindfulness Practice (HMP) was developed as a mobile-delivered approach in which brief guided exercises are linked to ordinary contexts such as commuting, eating, leisure activities, and household tasks. Rather than relying on a single structured session each day, HMP emphasizes multiple brief, cue-based practices distributed across routine activities. This design is consistent with recent arguments that digital technology may help extend meditation-based interventions by supporting practice in everyday contexts [12]. In HMP, ordinary routines are framed as recurring cues for brief mindfulness practice. This routine-embedded format is clinically relevant because mindfulness skills are intended to be applied during everyday experiences, including moments of stress, emotion, and interpersonal interaction [16,17]. By placing brief practices within familiar contexts, this format may make mindfulness easier to initiate and repeat when these skills are most relevant.

This design is informed by habit formation theory and research on contextual cueing [18], which suggest that repeated behaviors may become easier to initiate when they are consistently linked to stable contexts [19,20]. HMP extends this logic to mindfulness practice by treating recurring daily routines as cues for brief practices that may occur more often and with less initiation effort. This expectation is also consistent with evidence that brief mindfulness practices can improve distress and well-being, and that more regular practice is associated with better outcomes [21]. Thus, HMP represents a modification of how guided practice is organized in daily life, rather than a departure from the core principles of mindfulness [13,16].

Building on this rationale, this study evaluated a self-guided, mobile-delivered HMP intervention for adults with mild to moderate psychological distress. Primary outcomes were depressive and anxiety symptoms. Secondary outcomes included affective balance, adaptive and maladaptive emotion regulation, interpersonal difficulties, and mindfulness. HMP was compared with Traditional Mindfulness (TM), Mindfulness-Based Psychoeducation (MBP), and a waitlist control (WL). The active conditions addressed overlapping themes related to mindfulness, emotion, attention, and self-regulation, but differed in the role and organization of practice. MBP provided mindfulness-related psychoeducation without guided practice, TM paired comparable psychoeducational content with conventional structured practice, and HMP embedded brief guided practices within recurring daily activities. Outcomes were assessed at baseline (T1), immediately after the 21-day intervention (T2), and at a 3-month follow-up (T3).

The study had 3 aims. First, we tested whether the 2 practice-based conditions, HMP and TM, would lead to greater reductions in the confirmatory primary outcomes of depressive and anxiety symptoms than MBP and WL at postintervention. Second, we examined whether these practice-based conditions would also show more favorable changes in theoretically proximal secondary outcomes, including mindfulness and adaptive and maladaptive emotion regulation, as well as in broader exploratory outcomes, including affective balance and interpersonal difficulties. Third, given that HMP was designed to align practice more closely with everyday routines, we compared HMP with TM on engagement-related outcomes, including practice duration, attrition, and participant satisfaction. We expected HMP and TM to outperform MBP and WL on the primary outcomes and to show more favorable changes on secondary and exploratory outcomes, reflecting the role of guided practice beyond psychoeducational exposure. Given that HMP and TM shared the same therapeutic orientation but differed in practice structure, comparisons between these 2 mindfulness conditions were intended to evaluate delivery format rather than distinct treatment models. We therefore expected HMP and TM to show broadly comparable clinical benefits, while making an exploratory directional hypothesis that HMP would show more favorable engagement-related outcomes than TM.

## Methods

### Study Design and Registration

This study was a 4-arm parallel randomized controlled trial evaluating 2 self-guided mobile mindfulness interventions against an active psychoeducation condition and a WL. Participants were assigned in a 1:1:1:1 ratio to TM, HMP, MBP, or WL. The intervention lasted 21 days, with assessments conducted at baseline (T1), postintervention (T2), and a 3-month follow-up (T3). WL participants were offered access to intervention materials after postintervention, so no untreated WL follow-up data were collected. A sample size estimate using G*Power (version 3.1; Heinrich-Heine University Düsseldorf) indicated that 280 participants were required for the primary postintervention 4-group comparison, assuming a conservative small-to-medium effect size based on prior meta-analytic evidence on mindfulness apps [22], a Cohen *f* of 0.20, an α level of .05, a power of 0.80, and 4 groups. Given the high dropout rates commonly observed in self-guided digital mindfulness interventions [23], recruitment was increased to preserve sufficient data for the primary comparisons. The trial was approved by the Institutional Review Board (IRB) of the Faculty of Psychology at the affiliated university (IRB number BNU202404240092) and preregistered with the Chinese Clinical Trial Registry (ChiCTR2400093771). All procedures were completed online. The completed CONSORT-eHEALTH (Consolidated Standards of Reporting Trials of Electronic and Mobile Health Applications and Online Telehealth) checklist is provided in Checklist 1.

### Participants and Recruitment

Participants were adults aged 18 to 65 years who had smartphone access, were able to provide informed consent, and reported mild to moderate symptoms of anxiety or depression, defined as a score of 5 to 14 on the Generalized Anxiety Disorder–7 or Patient Health Questionnaire–9 [24-26]. Exclusion criteria included scores above 14 on either measure, current or planned psychological treatment, psychiatric medication use within the past 3 months, prior formal mindfulness training, a history of psychosis, or active suicidal ideation. These criteria were used to maintain the study’s focus on mild to moderate distress, reduce treatment-related confounding, and direct higher-risk participants toward more appropriate care. Recruitment was conducted through social media in August 2024, and all screening, consent, and baseline procedures were completed online. Individuals who were not eligible were provided with mental health support information, including crisis resources, and were encouraged to seek appropriate care when needed.

### Ethical Considerations

The study was approved by the IRB of the Faculty of Psychology at Beijing Normal University (IRB number BNU202404240092), China. All procedures were conducted in accordance with institutional ethical guidelines.

### Randomization and Masking

After the baseline assessment, participants were randomized by a computer-based procedure embedded in the study platform. Allocation was concealed from investigators before assignment. Participants were aware of their assigned materials after randomization, but they were not informed of the specific comparative hypotheses. Outcome data were collected online, and analysts were masked to group assignment during the primary analyses. No changes were made to the trial protocol or planned analyses after the study began.

### Intervention Platform and Procedures

All intervention materials were delivered through a mobile app. TM, HMP, and MBP participants received access to condition-specific materials, whereas WL participants completed assessments only during the 21-day study period and were offered intervention access afterward. The active conditions were comparable in delivery mode and daily task expectations but differed in whether and how guided practice was provided. App logs indexed exposure and access duration, although they could not verify practice fidelity. Additional intervention protocol details are provided in Multimedia Appendix 1.

Participants were asked to log in daily and complete at least 15 minutes of assigned content within each 24-hour window. TM involved one 15-minute guided mindfulness practice per day, HMP involved 3 routine-linked 5-minute guided practices per day, and MBP involved psychoeducational materials without guided practice. No monetary compensation was provided.

Outcome assessments were completed at baseline, postintervention, and 3-month follow-up. Follow-up analyses were limited to TM, HMP, and MBP because WL participants received intervention access after postintervention. No adverse events or unintended effects were reported during the study period.

### Intervention Conditions

#### Traditional Mindfulness

The TM condition was a 21-day, self-guided mobile mindfulness program based on practices commonly used in established mindfulness-based approaches [13-15]. Participants received standardized psychoeducational materials introducing core mindfulness principles, including present-moment awareness and nonjudgmental observation, followed by one 15-minute audio-guided practice each day. Seven practices were available: mindful breathing, body scan, seated meditation, mindful eating, mindful walking, mindful listening, and mindful stretching. These exercises were adapted for brief app-based delivery while retaining a structured, session-based format.

#### Habitual Mindfulness Practice

**Figure 1.**
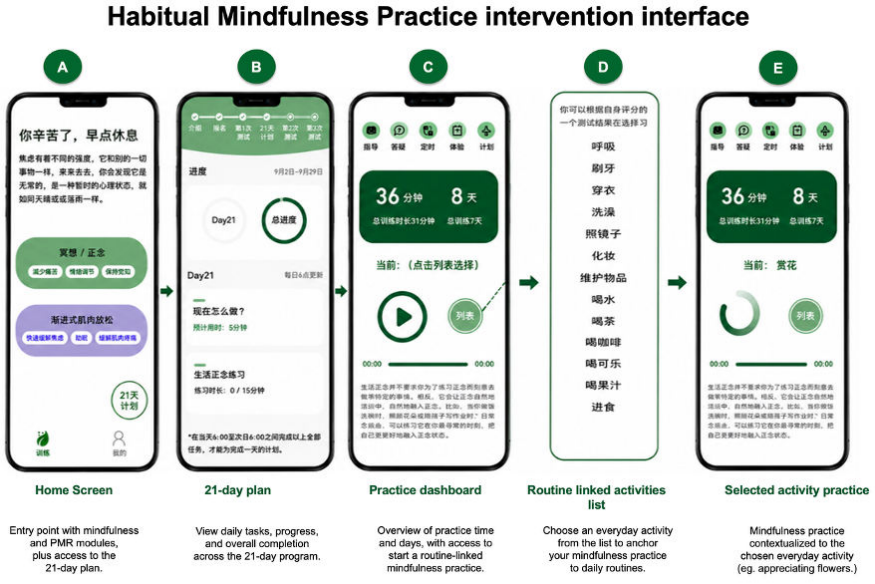
Overview of the Habitual Mindfulness Practice (HMP) intervention interface and guided practice flow. (A) Home screen, (B) 21-day plan, (C) practice dashboard, (D) routine linked activities list, and (E) selected activity practice. PMR: progressive muscle relaxation.

The HMP condition was a 21-day, self-guided mobile mindfulness program that embedded brief mindfulness exercises within recurring daily routines. Participants received the same psychoeducational materials as TM and completed 3 audio-guided 5-minute practices per day, totaling 15 minutes of daily practice. The practices were selected from 33 activities linked to common contexts, including eating or drinking, commuting, household tasks, leisure activities, and wellness-related routines. This format was designed to use recurring contexts as cues for brief practice rather than relying on one longer formal session. The audio-guided instructions were specifically developed for this study and refined through consultation with mindfulness experts. The HMP mobile app interface and intervention workflow are shown in Figure 1, illustrating the delivery structure of the intervention.

#### Mindfulness-Based Psychoeducation

The MBP condition served as an active comparison condition. Participants received the same psychoeducational materials on mindfulness concepts and principles as those in TM and HMP but did not complete guided mindfulness exercises. This condition was included to distinguish the effects of psychoeducational exposure from repeated mindfulness practice. Participants were asked to log in daily and engage with the assigned app-based materials during the 21-day intervention period.

#### Waitlist Control

Participants in the WL condition did not receive intervention materials during the 21-day study period but completed assessments through postintervention. After T2, they were offered access to the intervention materials; therefore, WL was not included in the T3 follow-up analyses.

### Outcomes and Measures

#### Overview

Assessments were completed at baseline (T1), immediately after the 21-day intervention period (T2), and 3 months after the end of the intervention (T3). The prespecified primary outcomes were depressive and anxiety symptoms. Secondary outcomes were mindfulness and cognitive emotion regulation; affective balance and interpersonal difficulties were considered exploratory outcomes. Engagement indicators were collected to evaluate intervention use and acceptability.

#### Primary Outcomes

Depressive symptoms were assessed using the Patient Health Questionnaire–9 [24]. Participants rated how often they had experienced each symptom during the previous 2 weeks on a 4-point scale ranging from 0 (“not at all”) to 3 (“nearly every day”). Total scores were calculated by summing item responses, with higher scores indicating greater depressive symptom severity. The Chinese version used in this study has shown acceptable reliability and validity in prior research [25]. Internal consistency in the present sample was high at all 3 assessments (T1 α=.86; T2 α=.91; T3 α=.89).

Anxiety symptoms were assessed using the Generalized Anxiety Disorder–7 [26]. Participants rated the frequency of core anxiety symptoms experienced during the previous 2 weeks on a 4-point scale ranging from 0 (“not at all”) to 3 (“nearly every day”). Total scores ranged from 0 to 21, with higher scores indicating greater anxiety symptom severity. The Chinese version has demonstrated acceptable psychometric performance in Chinese samples [20]. Internal consistency in the present sample was high across waves (T1 α=.79; T2 α=.83; T3 α=.77).

#### Secondary Outcomes

Mindfulness was assessed using the Chinese version of the Five Facet Mindfulness Questionnaire–Short Form [27,28]. This 20-item measure assesses 5 facets of dispositional mindfulness: observing, describing, acting with awareness, nonjudging of inner experience, and nonreactivity to inner experience. Items were rated on a 5-point scale ranging from 1 (“never”) to 5 (“always”). In this study, a total composite score was used to index overall mindfulness [29], with higher scores indicating greater dispositional mindfulness. Internal consistency was acceptable to good across time points (T1 α=.76; T2 α=.81; T3 α=.77).

Cognitive emotion regulation was assessed with the Cognitive Emotion Regulation Questionnaire [30]. The Chinese version used in this study includes 36 items covering 9 cognitive coping strategies used in response to stressful events [31]. Following prior scoring conventions, the strategies were grouped into adaptive and maladaptive emotion regulation indices. Participants responded on a 5-point scale ranging from 1 (“never”) to 5 (“always”). Higher scores indicated greater use of the corresponding type of strategy. Internal consistency was acceptable to good for both maladaptive regulation (T1 α=.73; T2 α=.79; T3 α=.82) and adaptive regulation (T1 α=.75; T2 α=.78; T3 α=.83).

#### Exploratory Outcomes

Affective balance was assessed with the Affective Balance Scale [32]. The measure includes 10 dichotomous items referring to positive and negative emotional experiences in the past few weeks. Consistent with the Chinese scoring approach used in this study [33], a positive affect score and a negative affect score were calculated separately, and an affect balance score was derived by subtracting negative affect from positive affect and adding a constant of 5. Higher scores indicated a more favorable affective balance. Internal consistency for the positive and negative affect subscales was modest to acceptable across assessments (positive affect: T1 α=.61; T2 α=.60; T3 α=.71; negative affect: T1 α=.60; T2 α=.77; T3 α=.72).

Interpersonal difficulties were assessed with the Interpersonal Comprehensive Diagnostic Scale [34], a 28-item self-report measure covering communication, friendship formation, social etiquette, and heterosexual interactions. Twenty-seven items were answered in a dichotomous format (1=“yes” to 0=“no”), and higher total scores indicated greater interpersonal difficulty. Internal consistency was good across all assessments (T1 α=.87; T2 α=.93; T3 α=.92).

#### Engagement Outcomes

Engagement with the intervention was evaluated using both app-recorded and self-reported indicators. App-recorded usage data were used to estimate practice duration and intervention exposure for the 3 active conditions. In addition, participants completed 6 study-specific engagement items assessing understanding of instructions, interest, discomfort, integration of practice into daily life, and continued practice after the intervention. Five items were averaged to create an overall engagement score, with higher scores indicating greater engagement. One open-ended item was retained for qualitative feedback but was not included in the quantitative analyses. Engagement comparisons focused mainly on HMP and TM because the study examined whether routine-embedded practice offered practical advantages over a session-based format.

### Statistical Analysis

All analyses were conducted using SPSS Statistics (version 27; IBM Corp) under an intention-to-treat framework. Descriptive statistics were examined at each assessment point. Baseline equivalence across the 4 groups was tested using ANOVA for continuous variables and chi-square tests for categorical variables.

Missing data were evaluated before outcome analyses. Little test suggested that missingness was consistent with missing completely at random (*P*=.12) [35,36]. Missing values were therefore estimated using the expectation-maximization algorithm. Given the substantial attrition, supplementary analyses compared completers and noncompleters on baseline clinical and psychological variables; no systematic baseline differences were found.

For postintervention outcomes at T2, between-group differences were examined using analyses of covariance, with baseline scores entered as covariates. This approach was used to compare the 4 randomized groups on the primary outcomes of depressive symptoms and anxiety symptoms, as well as on the secondary and exploratory outcomes. When the omnibus group effect was significant, pairwise comparisons were conducted with Bonferroni adjustment. Effect sizes are reported as partial ηp². To reduce the risk of inflated type I error across the primary postintervention tests, the Holm-Bonferroni procedure was applied.

To examine changes over time, longitudinal analyses were conducted using linear mixed-effects models. These models included fixed effects for group, time, and the group× time interaction, with a random intercept for participants and an unstructured covariance matrix. Because the WL group was not assessed at follow-up, the longitudinal models were estimated for the 3 active intervention conditions only: TM, HMP, and MBP. Post hoc comparisons for the mixed models were adjusted using the Sidak procedure. The mixed-model framework was used to characterize trajectories from baseline to postintervention and follow-up while accommodating repeated observations within individuals.

Engagement and acceptability analyses focused mainly on HMP and TM. Group differences in practice duration and self-reported engagement were tested using 2-tailed independent-samples *t* tests. Attrition-related comparisons used chi-square tests, *t* tests, and 2-way analyses of variance, as appropriate. All tests were 2-tailed, with statistical significance set at *P*<.05.

## Results

### Participant Flow and Baseline Characteristics

A total of 876 individuals were screened for eligibility. Of these, 190 were excluded because they did not meet the inclusion criteria (n=109), did not respond after screening (n=73), or were no longer interested in participating (n=8). The remaining 686 participants completed the baseline assessment and were randomized to TM (n=172), HMP (n=172), MBP (n=171), or WL (n=171). All 686 randomized participants were included in the intention-to-treat analyses.

**Figure 2.**
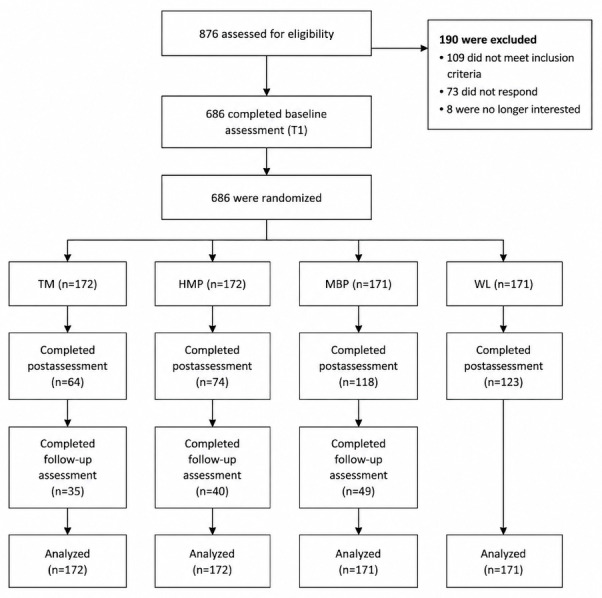
CONSORT (Consolidated Standards of Reporting Trials) diagram. This diagram illustrates the recruitment, randomization, and retention of participants across the 4 study groups. HMP: Habitual Mindfulness Practice; MBP: Mindfulness-Based Psychoeducation; TM: Traditional Mindfulness; WL: waitlist control.

Retention differed across conditions over time. At postintervention, outcome data were available for 64 participants in TM, 74 in HMP, 118 in MBP, and 123 in WL. At the 3-month follow-up, outcome data were available for 35 participants in TM, 40 in HMP, and 49 in MBP. As specified in the study design, no untreated follow-up data were collected for WL after participants were offered access to intervention materials. No adverse events or unintended effects were reported. Figure 2 presents the participant flow.

The 4 randomized groups were broadly comparable at baseline. The mean age ranged from 29.39 (SD 6.28) years to 30.68 (SD 7.97) years across conditions, and most participants had completed college education or higher. No significant between-group differences were observed for age, gender identity, educational attainment, or baseline scores on the primary and secondary outcomes.

### Postintervention Primary Outcomes

Observed means by group and assessment point are shown in Table 1. For the primary outcomes, ANCOVA controlling for baseline scores showed significant postintervention group effects for depressive symptoms (*F*_3,681_=28.67, *P*<.001, ηp²=0.11) and anxiety symptoms (*F*_3,681_=30.11, *P*<.001, ηp²=0.12). Both TM and HMP had lower depressive and anxiety symptom scores than MBP and WL. TM also showed lower anxiety than HMP (*P*=.04), whereas TM and HMP did not differ significantly on depressive symptoms (*P*=.63). Full Bonferroni-adjusted pairwise comparisons with *P* values are provided in the Detailed Postintervention Results section of Multimedia Appendix 2.

**Table 1.** Outcome means by group and assessment point.^a^

Outcome and assessment	TM^b^, mean (SD)	HMP^c^, mean (SD)	MBP^d^, mean (SD)	WL^e^, mean (SD)	ANCOVA
Depressive symptoms					*F* (3, 681)=28.67; *P*<.001; ηp²=0.11
T1	9.84 (4.69)	10.05 (4.97)	9.36 (4.68)	10.02 (4.81)	
T2	6.78 (3.86)	7.44 (3.59)	8.18 (4.39)	9.68 (5.38)	
T3	7.33 (2.92)	7.66 (3.73)	8.17 (4.71)	—^f^	
Anxiety symptoms					*F* (3, 681)=30.11; *P*<.001; ηp²=0.12
T1	9.07 (4.85)	8.78 (4.93)	7.97 (4.29)	9.04 (4.71)	
T2	5.57 (3.20)	6.28 (3.51)	7.21 (3.96)	8.29 (4.82)	
T3	6.05 (2.71)	6.24 (2.90)	7.54 (4.28)	—	
Interpersonal difficulties					*F* (3, 681)=19.50; *P*<.001; ηp²=0.08
T1	13.95 (6.14)	13.63 (6.25)	13.40 (6.47)	13.64 (5.80)	
T2	10.07 (5.48)	10.77 (5.51)	11.98 (6.47)	13.37 (6.64)	
T3	11.71 (5.65)	11.52 (5.76)	12.00 (6.46)	—	
Adaptive emotion regulation					*F* (3, 681)=6.85; *P*<.001; ηp²=0.03
T1	36.03 (9.11)	37.48 (9.35)	37.24 (9.29)	36.83 (8.18)	
T2	37.89 (5.93)	37.80 (5.27)	35.44 (6.74)	37.27 (8.34)	
T3	38.57 (9.06)	39.46 (8.06)	37.20 (9.74)	—	
Maladaptive emotion regulation					*F* (3, 681)=13.00; *P*<.001; ηp²=0.05
T1	32.11 (7.34)	32.94 (7.51)	31.36 (7.43)	33.44 (8.41)	
T2	31.18 (4.69)	31.21 (4.56)	30.02 (5.90)	34.29 (8.94)	
T3	29.19 (7.74)	30.27 (7.74)	30.71 (8.14)	—	
Mindfulness					*F* (3, 681)=20.88; *P*<.001; ηp²=0.08
T1	56.88 (9.30)	57.06 (9.68)	56.80 (9.88)	55.98 (9.06)	
T2	63.46 (9.20)	63.45 (9.18)	59.02 (9.65)	58.01 (9.56)	
T3	61.82 (7.59)	61.93 (7.89)	59.73 (8.45)	—	
Affective balance					*F* (3, 681)=12.03; *P*<.001; ηp²=0.05
T1	3.76 (2.04)	4.04 (2.07)	4.19 (2.34)	3.70 (2.20)	
T2	5.37 (1.83)	5.23 (1.55)	4.52 (1.89)	4.56 (2.01)	
T3	5.12 (1.44)	5.13 (1.46)	4.53 (1.89)	—	

a Values are observed means (SDs). ANCOVA results refer to postintervention group effects controlling for baseline scores. Pairwise comparisons are provided in Multimedia Appendix 2. WL was not assessed at follow-up.

b TM: Traditional Mindfulness.

c HMP: Habitual Mindfulness Practice.

d MBP: Mindfulness-Based Psychoeducation.

e WL: waitlist control.

f Not applicable.

### Postintervention Secondary and Exploratory Outcomes

For secondary and exploratory outcomes, significant postintervention group effects were observed for interpersonal difficulties (*F*_3,681_=19.50, *P*<.001, ηp²=0.08), adaptive emotion regulation (*F*_3,681_=6.85, *P*<.001, ηp²=0.03), maladaptive emotion regulation (*F*_3,681_=13.00, *P*<.001, ηp²=0.05), mindfulness (*F*_3,681_=20.88, *P*<.001, ηp²=0.08), and affective balance (*F*_3,681_=12.03, *P*<.001, ηp²=0.05). TM and HMP showed more favorable scores than MBP and WL on mindfulness, affective balance, and interpersonal difficulties. For adaptive emotion regulation, MBP showed lower scores than TM, HMP, and WL. For maladaptive emotion regulation, WL showed higher scores than all 3 active intervention conditions. Full Bonferroni-adjusted pairwise comparisons are provided in Multimedia Appendix 2.

### Longitudinal Outcomes

Longitudinal analyses were conducted for TM, HMP, and MBP only because the WL group was not assessed at follow-up. For depressive symptoms, mixed-effects models showed a significant effect of time (*F*_2,515_=117.51, *P*<.001, ηp²=0.31) and a significant group × time interaction (*F*_4,515_=7.74, *P*<.001, ηp²=0.06). For anxiety symptoms, the effect of time (*F*_2,515_=97.90, *P*<.001, ηp²=0.28) and the group × time interaction (*F*_4,515_=13.07, *P*<.001, ηp²=0.09) were also significant. TM and HMP maintained lower depressive symptom scores than MBP at postintervention and follow-up, with no significant difference between TM and HMP. For anxiety symptoms, both mindfulness conditions showed lower scores than MBP at postintervention and follow-up; TM showed lower anxiety than HMP at postintervention, but this difference was not significant at follow-up.

Secondary and exploratory outcomes showed a more variable maintenance pattern. Significant group × time interactions were observed for adaptive emotion regulation (*F*_4,515_=7.74, *P*<.001), maladaptive emotion regulation (*F*_4,515_=3.10, *P*=.02), mindfulness (*F*_4,515_=13.86, *P*<.001), interpersonal difficulties (*F*_4,515_=17.11, *P*<.001), and affective balance (*F*_4,515_=8.02, *P*<.001). Affective balance showed relatively sustained improvement, whereas mindfulness and interpersonal difficulties declined somewhat after postintervention while remaining improved relative to baseline. Emotion regulation outcomes also showed evidence of change over time, with stronger patterns for the mindfulness practice conditions than for MBP on maladaptive regulation. Full mixed-effects model statistics and post hoc comparisons are provided in Multimedia Appendix 2.

### Engagement and Attrition

Attrition was substantial in both mindfulness practice conditions. At postintervention, outcome data were unavailable for 62.8% (108/172) of participants in TM and 57% (98/172) in HMP, compared with 31% (53/171) in MBP and 28.1% (48/171) in WL. Among participants assigned to the active conditions, 59.3% (102/172) in TM, 65.1% (112/172) in HMP, and 71.3% (122/171) in MBP completed at least 10 intervention days.

HMP did not show a clear engagement advantage over TM. Total practice duration, self-reported engagement, and participant satisfaction did not differ significantly between the 2 mindfulness practice conditions. Supplementary attrition analyses showed no significant differences between completers and noncompleters in age or other baseline clinical and psychological variables, and no significant group × completion status interactions were observed. Detailed attrition analyses, including age comparisons, are provided in Tables S2 and S3 in Multimedia Appendix 2.

## Discussion

### Principal Findings

This randomized controlled trial examined whether a routine-embedded mobile mindfulness format could reduce psychological distress in adults with mild to moderate symptoms of anxiety or depression and whether this format offered practical advantages over a more traditional app-delivered mindfulness program. Three findings deserve emphasis. First, both mindfulness practice conditions, HMP and TM, showed greater postintervention reductions in depressive symptoms than MBP and WL. Second, both mindfulness conditions also showed lower anxiety than MBP and WL, although TM produced a somewhat stronger short-term effect on anxiety than HMP. Third, the 2 mindfulness conditions showed more favorable outcomes than the comparison conditions on several secondary measures, including mindfulness, cognitive emotion regulation, affective balance, and interpersonal difficulties. These findings are consistent with prior evidence that MBIs can reduce symptoms of anxiety and depression [10,11] and can also influence broader aspects of emotional functioning [6-8].

These findings suggest that repeated mindfulness practice, rather than psychoeducational exposure alone, was associated with broader improvements in emotional functioning and distress-related outcomes in this sample. HMP was associated not only with reductions in depressive and anxiety symptoms but also with improvements in affective balance and interpersonal functioning. Because affective balance reflects the relative predominance of positive over negative emotional states [32,33], improvements in this domain may indicate shifts in the overall emotional tone of everyday life. Reductions in interpersonal difficulties further suggest potential benefits in relational functioning, consistent with prior work linking mindfulness to interpersonal processes [7,8]. Together, these results indicate that the value of HMP may extend beyond symptom reduction to broader aspects of daily experience.

The findings also align with the study’s expectation that HMP and TM would show broadly comparable clinical benefits, although with one important qualification. HMP and TM did not differ significantly in depressive symptoms at postintervention, and both conditions showed more favorable outcomes than MBP and WL across several outcomes. For anxiety, TM showed lower scores than HMP immediately after the intervention, but this difference was not observed at follow-up. This finding may reflect the different practice structures used in the 2 conditions. TM involved one continuous 15-minute practice, whereas HMP divided the same total daily practice time into three shorter routine-linked practices. Prior work comparing formal and informal mindfulness formats similarly suggests that both formats can be beneficial, but that informal or daily-life-integrated practice is not necessarily superior to formal practice [37]. A continuous practice period may provide more opportunity for sustained attention and settling during a single episode, which may be especially relevant for short-term anxiety symptoms. Given that the between-condition difference was limited to postintervention, this finding should be interpreted cautiously. The present findings therefore suggest that HMP is a viable routine-embedded format, while also indicating that changing the structure of practice does not necessarily produce stronger immediate anxiety effects than a session-based format.

The longitudinal findings provide some support for the durability of these effects, although the pattern was not uniform across outcomes. Improvements in depressive symptoms were largely maintained at follow-up, and both mindfulness conditions continued to show more favorable trajectories than MBP. Anxiety remained lower than baseline, although the temporal pattern differed somewhat between conditions. For secondary outcomes, maintenance was more variable, with affective balance showing relatively sustained improvement, whereas mindfulness and interpersonal difficulties showed some decline over time.

The exploratory expectation that HMP would show more favorable engagement-related outcomes than TM was not supported. Although HMP was designed to reduce friction in practice initiation and improve practical fit with daily routines, HMP did not differ significantly from TM in practice duration, attrition, or participant satisfaction. This suggests that restructuring practice around daily routines may help preserve clinical benefit but may not be sufficient on its own to overcome the broader engagement challenges common in self-guided digital interventions. Engagement may also depend on motivational and accountability supports, including feedback, reminders, brief guidance, or peer- and group-based practice [38,39]. A more interactive or blended version of HMP could therefore be examined in future work as a way to strengthen adherence while preserving the accessibility and routine-embedded design of the intervention.

### Comparison With Prior Work

The present findings are broadly consistent with prior work suggesting that digitally delivered mindfulness interventions can reduce anxiety and depressive symptoms [9-11] and can also influence broader aspects of emotional functioning [5,33]. What this study adds is a more direct comparison between 2 app-delivered mindfulness formats that differed not in general therapeutic orientation but in how practice was structured in daily life.

Much of the digital mindfulness literature has focused on whether app-based mindfulness is beneficial relative to control conditions [11,15]. This study addressed a somewhat different question: whether changing the delivery structure of practice, from one longer formal session to multiple brief routine-linked practices, might preserve benefit while better fitting the realities of everyday use. In that respect, the findings are encouraging but qualified. HMP showed broadly comparable benefits to TM across most outcomes, although TM showed a short-term advantage for anxiety at postintervention. This suggests that mindfulness practice need not be restricted to a single formal daily session to remain useful, while also indicating that routine-linked practice should not be assumed to be superior to session-based practice. Brief, context-linked exercises may still support symptom improvement and broader emotional functioning. This is consistent with work emphasizing the integration of mindfulness into ordinary activities rather than restricting practice to formal sessions alone [16,40]. In this sense, HMP may be understood not as a simplified version of mindfulness, but as a differently organized practice format designed to fit more naturally into everyday life.

At the same time, the findings do not indicate that HMP conferred a clear advantage over TM, either in clinical outcomes or engagement. The contribution of the study is, therefore, less about demonstrating superiority and more about showing that a routine-embedded format can remain viable without a loss of efficacy across most outcomes.

Another useful comparison concerns the psychoeducation-only condition. MBP showed a weaker and less consistent pattern of effects than the 2 mindfulness practice conditions. This pattern is consistent with the view that psychoeducational exposure alone may be less likely to produce broader changes in emotional functioning when it is not accompanied by repeated experiential practice [41,42]. In this trial, MBP did not match the mindfulness conditions on the primary outcomes and was generally less favorable on the process-related outcomes as well. This does not imply that psychoeducation is unhelpful, but suggests that conceptual knowledge alone is unlikely to account for the observed improvements.

### Limitations

Several limitations should be acknowledged. First, attrition was substantial, particularly in the 2 mindfulness practice conditions. Although the intention-to-treat analyses and the baseline comparisons between completers and noncompleters reduce concern that the findings were driven entirely by selective retention, the level of dropout still places an important constraint on interpretation. In particular, the engagement promise of HMP remains unresolved because the routine-embedded format did not clearly reduce attrition relative to TM.

Second, engagement was assessed using app-recorded usage and brief self-report indicators, neither of which can fully capture the quality or fidelity of mindfulness practice. App logs indexed exposure to the intervention materials, but they could not establish whether participants completed every exercise as intended or whether some routine-based practice occurred outside the tracked platform. This is especially relevant for HMP because its structure may make off-platform practice more likely. As a result, the present engagement indicators should be interpreted as approximations rather than precise measures of actual mindfulness practice.

Third, the WL group was not followed beyond postintervention because participants in that group were offered access to the intervention materials after T2. This limits the strength of conclusions about longer-term comparative effects. The follow-up analyses are still informative for describing the trajectories of the 3 active conditions, but they do not allow firm statements about whether the observed maintenance effects would remain stronger than no intervention over the same period.

Fourth, the sample consisted of adults with mild to moderate self-reported distress recruited online, rather than participants selected on the basis of diagnostic interviews or treatment-seeking clinical status. The illness duration, time since diagnosis, and prior treatment history were not assessed, precluding examination of whether attrition differed by the chronicity of mental health difficulties. The findings, therefore, speak most directly to low-intensity digital intervention use in individuals with elevated distress, not to more severe or complex clinical presentations. This limits generalizability to higher-risk populations and to settings in which self-guided app use is not the primary mode of delivery. Future studies should collect more detailed clinical history, as prior research suggests that treatment history and clinical severity may be relevant to dropout [43,44].

Finally, although HMP was conceptually informed by habit formation and contextual cueing [18-20], this study did not directly measure habit strength, automaticity, or cue-response consistency. For that reason, the study can show that a routine-embedded format was feasible and beneficial, but it cannot demonstrate that habit formation itself was the mechanism responsible for the observed effects. Future work would benefit from including more direct assessments of routine linkage, cue-dependent initiation, and sustained practice over time.

### Conclusions

In summary, this trial provides evidence that a routine-embedded, self-guided mobile mindfulness intervention may help reduce mild to moderate psychological distress. HMP and TM both outperformed psychoeducation alone and WL on depressive and anxiety symptoms and showed broader benefits across several measures of emotional functioning. However, HMP did not show better adherence, greater satisfaction, or stronger short-term efficacy than TM. The main implication is, therefore, not that routine embedding is superior to traditional app-delivered mindfulness, but that it may represent a viable alternative way of delivering mindfulness practice in digital mental health settings. This may be useful for future intervention design, particularly if combined with stronger strategies to support retention and continued engagement.

## Supplementary material

10.2196/98056 Multimedia Appendix 1 Supplementary intervention protocol.

10.2196/98056 Multimedia Appendix 2 Supplementary results.

10.2196/98056 Checklist 1 CONSORT-eHEALTH checklist (V1.6.1).

## Abbreviations

CONSORT-eHEALTH: Consolidated Standards of Reporting Trials of Electronic and Mobile Health Applications and Online Telehealth
HMP: Habitual Mindfulness Practice
IRB: Institutional Review Board
MBI: mindfulness-based intervention
MBP: Mindfulness-Based Psychoeducation
TM: Traditional Mindfulness
WL: waitlist control
