# Supplementary material for: A Self-Guided Mobile Mindfulness Intervention Embedded in Daily Routines for Adults With Mild to Moderate Psychological Distress: Randomized Controlled Trial
Source: JMIR Ment Health. 2026 Jul 15;13:e98056. doi: 10.2196/98056 (PMC13372293; doi:10.2196/98056)
Supplement: Multimedia Appendix 1 [file mental-v13-e98056-s001.docx]

**Multimedia Appendix 2. Intervention Protocols**

**Overview of Study Conditions**

Four conditions were included in the trial: Habitual Mindfulness Practice (HMP), Traditional Mindfulness (TM), Mindfulness-Based Psychoeducation (MBP), and a waitlist control (WL). The three active conditions shared a common psychoeducational framework addressing mindfulness, emotion, attention, and self-regulation. However, the framing and delivery of practice differed across conditions. TM emphasized one structured guided practice session per day, whereas HMP emphasized multiple brief practices linked to naturally recurring daily contexts. MBP received psychoeducational materials only and did not include guided mindfulness exercises.

**Table S1. Psychoeducational Framework for Habitual Mindfulness Practice (HMP)**

| **Module** | **Topic** | **Key focus** |
| --- | --- | --- |
| 1 | Introduction to habitual mindfulness practice | Introduced mindfulness as present-moment, nonjudgmental awareness. Framed mindfulness as something that can be embedded within ordinary routines rather than limited to designated sessions. Emphasized the use of naturally recurring daily activities as cues for brief mindful attention. |
| 2 | Understanding emotions | Introduced emotions as involving subjective experience, physiological arousal, and behavioral expression. Encouraged noticing emotional responses during ordinary interactions and routine situations. |
| 3 | Disengaging from automatic thinking | Introduced the idea of cognitive “autopilot” and encouraged noticing habitual reactions during everyday activities. Emphasized pausing and returning attention to the present when automatic thinking became dominant. |
| 4 | Embodied presence | Encouraged attention to bodily sensations as a way of grounding awareness in the present moment. Framed ordinary activities as opportunities to reconnect attention with the body. |
| 5 | Returning to the present | Addressed mind-wandering and the tendency to become caught in past- or future-oriented thinking. Encouraged brief redirection of attention to immediate sensory experience during routine activities. |
| 6 | Recognizing avoidance responses | Addressed avoidance as a common response to discomfort and encouraged participants to notice rather than automatically resist difficult internal experiences. |
| 7 | Allowing and letting be | Introduced acceptance and non-resistance to present-moment experience. Encouraged participants to remain with difficult thoughts or feelings without immediately trying to change them. |
| 8 | Thoughts are not facts | Introduced cognitive distancing from thoughts and encouraged participants to notice thoughts as mental events rather than as objective facts. |
| 9 | Cultivating self-compassion | Introduced self-compassion as a less self-critical way of responding to distress. Encouraged brief compassionate awareness during setbacks or emotionally difficult moments. |
| 10 | Sustaining mindful living | Emphasized maintaining mindfulness through small, repeated routine-based practices. Encouraged participants to continue using daily cues to support ongoing mindful awareness after the intervention period. |

Note. The HMP psychoeducational materials emphasized embedding mindfulness into naturally recurring daily contexts and using those contexts as prompts for brief, repeated practice.

**Table S2. Practical Practice Components for Habitual Mindfulness Practice (HMP)**

| **Practice theme** | **Example activities** |
| --- | --- |
| Daily routines | Breathing; brushing teeth; dressing; showering; looking in the mirror; applying makeup; applying skincare |
| Eating and drinking | Drinking water; drinking tea; drinking coffee; drinking juice; having a meal; eating fruit; eating chocolate; eating biscuits/cookies |
| Commuting and movement | Walking; riding the bus; taking the subway |
| Versatile/other situations | Standing; resting while sitting |
| Wellness and exercise | Foot soaking; running; pre-sleep relaxation |
| Household chores | Sweeping the floor |
| Leisure | Petting a cat; watering plants; gazing at the sky; watching the sunset; observing scenery; observing crowds; admiring flowers |

Note. Participants in the HMP condition completed three 5-minute audio-guided practices per day over 21 days, for a total planned practice time of 315 minutes. Practices were designed to be completed during naturally occurring daily activities rather than in a separate formal practice period.

**Table S3. Traditional Mindfulness (TM): Psychoeducational Framework and Practice Protocol**

**A. Psychoeducational framework**

| **Module** | **Topic** | **Key focus** |
| --- | --- | --- |
| 1 | Introduction to mindfulness | Introduced mindfulness as present-moment, nonjudgmental awareness, with emphasis on structured guided practice sessions. Oriented participants to the role of dedicated practice periods in cultivating mindful attention. |
| 2–9 | Core psychoeducational themes | Addressed the same broad themes as the HMP condition, including emotions, automatic thinking, embodied awareness, present-moment attention, avoidance, acceptance, cognitive distancing, and self-compassion. In TM, these themes were paired with session-based guided practice rather than routine-based cueing. |
| 10 | Sustaining mindful practice | Emphasized maintaining mindfulness through continued structured practice, with attention to consistency, resistance to practice, and ongoing engagement with formal guided exercises. |

Note. TM and HMP shared overlapping psychoeducational themes, but differed in how practice was framed and implemented. TM emphasized dedicated guided sessions, whereas HMP emphasized cue-based practice embedded in daily routines.

**B. Practical practice protocol for Traditional Mindfulness (TM)**

| **Component** | **Description** |
| --- | --- |
| Objective | To deliver structured, session-based mindfulness training through guided formal exercises. |
| Format | Participants completed one 15-minute audio-guided practice per day over 21 days, together with psychoeducational readings. |
| Core exercises | Seven guided practices were available: mindful breathing; body scan; seated meditation; mindful eating; mindful walking; mindful listening; and mindful stretching. |
| Delivery | Audio-guided practices were adapted from established mindfulness-based approaches for use in a digital, self-guided format. Psychoeducational materials addressed core mindfulness concepts, emotional awareness, and self-regulation. |

**Table S4. Mindfulness-Based Psychoeducation (MBP): Protocol**

| **Component** | **Description** |
| --- | --- |
| Objective | To isolate the effects of mindfulness-related psychoeducational exposure from the effects of guided mindfulness practice. |
| Format | Approximately 15 minutes of psychoeducational reading per day for 21 days. |
| Content | Core psychoeducational themes related to mindfulness, emotional awareness, attention, and self-regulation. |
| Practice | No formal or informal guided mindfulness exercises. |
| Delivery | Text-based psychoeducational materials delivered through the mobile platform. |

Note. MBP was designed as a psychoeducation-only comparison condition.

**Waitlist Control (WL)**

Participants assigned to the WL condition did not receive intervention materials during the 21-day study period. They completed assessments on the same schedule as the intervention groups through post-intervention assessment and were offered access to intervention materials afterward.

## References

No references cited.
