# Supplementary material for: A Self-Guided Mobile Mindfulness Intervention Embedded in Daily Routines for Adults With Mild to Moderate Psychological Distress: Randomized Controlled Trial
Source: JMIR Ment Health. 2026 Jul 15;13:e98056. doi: 10.2196/98056 (PMC13372293; doi:10.2196/98056)
Supplement: Multimedia Appendix 2 [file mental-v13-e98056-s002.docx]

**Multimedia Appendix 1. Supplementary Results**

**Abbreviations**

PHQ-9 = Patient Health Questionnaire-9 (depression)

GAD-7 = Generalized Anxiety Disorder-7 (anxiety)

FFMQ-SF = Five Facet Mindfulness Questionnaire–Short Form (mindfulness)

ICDS = Interpersonal difficulties were assessed with the Interpersonal Comprehensive Diagnostic Scale (ICDS)

CERQ = Cognitive Emotion Regulation Questionnaire

ABS = Affective Balance Scale

TM = Traditional Mindfulness

HMP=Habitual Mindfulness Practice

MBP=Mindfulness-Based Psychoeducation

WL = Waitlist Control

T1 = Baseline

T2 = Post-intervention (21 days)

T3 = Follow-up (3 months)

**Table S1. Baseline Demographic Characteristics by Group**

| Characteristic | TM (n=172) | HMP (n=172) | MBP (n=171) | WL (n=171) |
| --- | --- | --- | --- | --- |
| Age, mean (SD) | 29.95 (8.25) | 29.63 (6.28) | 30.45 (7.91) | 30.68 (7.97) |
| Gender identity, n (%) |  |  |  |  |
| Female | 157 (91.3) | 157 (91.3) | 153 (89.5) | 150 (87.7) |
| Male | 12 (7.0) | 15 (8.7) | 15 (8.8) | 21 (12.3) |
| Other gender | 3 (1.7) | 0 (0.0) | 0 (0.0) | 0 (0.0) |
| Education level, n (%) |  |  |  |  |
| High school or below | 6 (3.5) | 7 (4.1) | 6 (3.6) | 9 (5.3) |
| College or above | 166 (96.5) | 165 (95.9) | 165 (96.5) | 162 (94.7) |

Note. TM = Traditional Mindfulness; HMP=Habitual Mindfulness Practice; MBP=Mindfulness-Based Psychoeducation; WL = Waitlist Control.

Baseline demographic characteristics were comparable across TM, HMP, MBP, and WL, indicating successful randomization.

**Table S2. Baseline Differences Between Completers and Dropouts**

| Measure | Dropouts, mean (SD) (n=303) | Completers, mean (SD) (n=382) | t | *P* value | Mean difference | 95% CI |
| --- | --- | --- | --- | --- | --- | --- |
| Age | 30.47 (7.50) | 30.54 (8.17) | -0.11 | .913 | -0.07 | [-1.25, 1.12] |
| PHQ-9 | 9.98 (4.75) | 9.67 (4.82) | 0.83 | .409 | 0.30 | [-0.42, 1.03] |
| ABS | 3.82 (2.13) | 3.96 (2.21) | -0.82 | .413 | -0.14 | [-0.47, 0.19] |
| FFMQ-SF | 56.27 (9.82) | 57.00 (9.21) | -1.00 | .317 | -0.73 | [-2.16, 0.70] |
| GAD-7 | 9.02 (4.84) | 8.41 (4.60) | 1.70 | .090 | 0.61 | [-0.10, 1.33] |
| ICDS | 13.76 (6.32) | 13.28 (6.00) | 1.02 | .310 | 0.48 | [-0.45, 1.41] |
| CERQ adaptive | 37.10 (9.85) | 36.71 (8.26) | 0.56 | .576 | 0.39 | [-0.97, 1.75] |
| CERQ maladaptive | 32.95 (7.94) | 32.07 (7.51) | 1.49 | .137 | 0.88 | [-0.28, 2.05] |

*Note. CI = confidence interval; PHQ-9 = Patient Health Questionnaire-9; ABS = Affective Balance Scale; FFMQ-SF = Five Facet Mindfulness Questionnaire–Short Form; GAD-7 = Generalized Anxiety Disorder-7; ICDS = Interpersonal Comprehensive Diagnostic Scale; CERQ = Cognitive Emotion Regulation Questionnaire. Independent-samples t tests indicated no significant baseline differences between completers and dropouts on age or the study variables listed in the table (all P>.05).*

Independent-samples t tests indicated no significant baseline differences between completers and dropouts on age or the study variables listed in Table S2 (all *P*> .05).

**Table S3. Baseline Characteristics by Intervention Group and Completion Status**

| Measure | Group | Completers, mean (SD) | Dropouts, mean (SD) | F status | F interaction |
| --- | --- | --- | --- | --- | --- |
| Age | TM | 30.05 (9.25) | 29.90 (7.68) | 0.46 | 0.86 |
|  | HMP | 29.46 (6.75) | 29.76 (5.94) |  |  |
|  | MBP | 31.97 (8.49) | 31.36 (9.07) |  |  |
|  | WL | 30.07 (7.95) | 32.25 (7.89) |  |  |
| PHQ-9 | TM | 9.19 (4.70) | 10.24 (4.68) | 0.31 | 1.09 |
|  | HMP | 9.66 (4.78) | 10.37 (5.12) |  |  |
|  | MBP | 9.58 (4.78) | 8.89 (4.46) |  |  |
|  | WL | 10.02 (4.97) | 9.81 (4.37) |  |  |
| ABS | TM | 3.89 (2.18) | 3.61 (1.97) | 0.56 | 1.28 |
|  | HMP | 3.86 (1.92) | 4.13 (2.20) |  |  |
|  | MBP | 4.33 (2.43) | 3.72 (2.10) |  |  |
|  | WL | 3.69 (2.14) | 3.79 (2.34) |  |  |
| FFMQ-SF | TM | 57.91 (9.46) | 56.24 (9.25) | 1.62 | 0.15 |
|  | HMP | 57.48 (9.13) | 56.72 (10.14) |  |  |
|  | MBP | 57.18 (9.90) | 55.94 (9.91) |  |  |
|  | WL | 56.05 (8.45) | 55.79 (10.57) |  |  |
| CERQ adaptive | TM | 34.16 (8.86) | 37.07 (9.12) | 0.60 | 1.31 |
|  | HMP | 37.73 (7.74) | 37.28 (10.52) |  |  |
|  | MBP | 37.44 (8.98) | 36.79 (10.03) |  |  |
|  | WL | 36.71 (7.29) | 37.15 (10.18) |  |  |
| CERQ maladaptive | TM | 31.39 (7.06) | 32.49 (7.52) | 2.64 | 0.09 |
|  | HMP | 32.44 (6.63) | 33.35 (8.16) |  |  |
|  | MBP | 31.19 (7.47) | 31.74 (7.39) |  |  |
|  | WL | 33.02 (8.20) | 34.52 (8.91) |  |  |
| GAD-7 | TM | 8.27 (4.68) | 9.48 (4.92) | 1.85 | 0.39 |
|  | HMP | 8.55 (4.52) | 8.97 (5.27) |  |  |
|  | MBP | 7.90 (4.34) | 8.13 (4.24) |  |  |
|  | WL | 8.88 (4.85) | 9.08 (4.38) |  |  |
| ICDS | TM | 13.36 (6.63) | 13.30 (6.48) | 1.09 | 0.79 |
|  | HMP | 12.81 (5.57) | 14.29 (6.71) |  |  |
|  | MBP | 13.08 (5.98) | 14.09 (6.13) |  |  |
|  | WL | 13.74 (5.97) | 13.37 (5.41) |  |  |

Note. TM = Traditional Mindfulness; HMP=Habitual Mindfulness Practice; MBP=Mindfulness-Based Psychoeducation; WL = Waitlist Control. F status tests completer versus dropout differences; F interaction tests group × completion status interactions. All reported F values were nonsignificant (*P*>.05).

Two-way ANOVAs (group × completion status) showed no significant main effects of completion status or group × completion status interactions across age and baseline clinical and psychological measures, indicating that attrition was not systematically associated with participants’ baseline characteristics.

**Detailed Postintervention Results**

For depressive symptoms (PHQ-9), there was a significant main effect of group, F(3,681)=28.67, *P*<.001, ηp²=.11. Bonferroni-adjusted pairwise comparisons indicated that both TM and HMP had significantly lower depression scores than MBP and WL (all *P*<.01). MBP also showed lower scores than WL (*P*=.006), whereas no significant difference was observed between TM and HMP (*P*=.632).

For anxiety symptoms (GAD-7), a significant group effect was observed, F(3,681)=30.11, P<.001, ηp²=.12. TM showed significantly lower anxiety scores than HMP (*P*=.042), MBP, and WL (all *P*<.001). HMP also showed lower anxiety scores than MBP and WL (both *P*<.001), whereas MBP and WL did not differ significantly (*P*=.746).

For interpersonal difficulties(ICDS), there was a significant main effect of group, F(3,681)=19.50, *P*<.001, ηp²=.08. Both TM and HMP had significantly lower scores than MBP and WL (all *P*<.05), with no significant difference between TM and HMP (*P*=.321).

For adaptive emotion regulation, a significant group effect was observed, F(3,681)=6.85, *P*<.001, ηp²=.03. MBP showed significantly lower adaptive regulation than TM (*P*<.001), HMP (*P*=.004), and WL (*P*=.020), with no other significant differences.

For maladaptive emotion regulation, there was a significant group effect, F(3,681)=13.00, *P*<.001, ηp²=.05. The WL group reported higher maladaptive regulation than TM, HMP, and MBP (all *P*<.001), whereas no differences were observed among the three intervention groups (all *P*>.05).

For mindfulness (FFMQ-SF), a significant group effect was found, F(3,681)=20.88, *P*<.001, ηp²=.08. Both TM and HMP showed higher scores than MBP and WL (all *P* <.001), with no significant difference between TM and HMP (*P*=1.00).

For affective balance, a significant group effect was observed, F(3,681)=12.03, *P*<.001, ηp²=.05. Both TM and HMP showed higher scores than MBP and WL (all P<.001), whereas no significant differences were found between TM and HMP (*P*=.52) or between MBP and WL (*P*=1.00).

### Longitudinal Results

For depressive symptoms, the linear mixed-effects model showed a significant main effect of time, F(2,515)=117.51, *P*<.001, ηp²=.31, and a significant group by time interaction, F(4,515)=7.74, *P*<.001, ηp²=.06. The main effect of group was not significant, F(2,515)=1.19, P=.306. Sidak-adjusted comparisons indicated significant reductions from baseline to postintervention (*P*<.001) and from baseline to follow-up (*P* <.001), with no significant change from postintervention to follow-up (*P*=.120). MBP showed significantly higher depressive symptom scores than both TM and HMP at postintervention and follow-up (all *P*<.05), whereas no differences were observed between TM and HMP (*P*>.05).

For anxiety symptoms, the linear mixed-effects model showed a significant main effect of time, F(2,515)=97.90, *P*<.001, ηp²=.28, and a significant group by time interaction, F(4,515)=13.07, *P*<.001, ηp²=.09. The main effect of group was not significant, F(2,515)=1.90, *P*=.150. Anxiety decreased from baseline to postintervention (*P*<.001) and remained lower at follow-up (P<.001), with a marginal change from postintervention to follow-up (*P* =.054). Both TM and HMP showed lower anxiety scores than MBP at postintervention and follow-up (all *P*<.05). TM also showed lower anxiety than HMP at postintervention (*P*<.05), whereas this difference was not significant at follow-up (*P* >.05).

For adaptive emotion regulation, the linear mixed-effects model showed a significant main effect of time, F(2,515)=7.72, *P*<.001, ηp²=.02, and a significant group by time interaction, F(4,515)=7.74, *P*<.001. Scores increased from baseline to follow-up (*P*≤.002), with no change from baseline to postintervention (*P*=.984). HMP showed higher scores than MBP at postintervention (*P*=.049), with no other significant between-group differences.

For maladaptive emotion regulation, there was a significant main effect of time, F(2,515)=17.68, *P*<.001, ηp²=.06, and a significant group by time interaction, F(4,515)=3.10, *P* =.015. Scores decreased from baseline to postintervention (*P* <.001) and follow-up (*P*<.001), with no significant change between postintervention and follow-up (*P*=.083). TM and HMP showed greater reductions than MBP across time (all *P*<.05).

For mindfulness (FFMQ-SF), significant main effects of group, F(2,515)=4.02, *P*=.019, ηp²=.02, and time, F(2,515)=193.66, *P*<.001, ηp²=.43, were observed, along with a significant interaction, F(4,515)=13.86, *P*<.001. Both TM and HMP had higher scores than MBP ( =.049 and P=.037, respectively), with no difference between TM and HMP (*P*=1.00). Scores increased from baseline to postintervention and follow-up (both *P* <.001) and decreased from postintervention to follow-up (*P*<.001).

For interpersonal difficulties (ICDS), there was a significant main effect of time, F(2,515)=168.58, *P*<.001, ηp²=.40, and a significant interaction, F(4,515)=17.11, *P*<.001. Scores decreased from baseline to postintervention (*P*<.001) and follow-up (P<.001), with an increase from postintervention to follow-up (*P*<.001). No significant group differences were observed (all *P* >.75).

For affective balance, significant main effects of group, F(2,515)=3.45, *P*=.032, ηp²=.01, and time, F(2,515)=64.52, *P*<.001, ηp²=.20, were observed, along with a significant interaction, F(4,515)=8.02, *P*<.001. HMP showed higher scores than MBP (*P* =.048), with no other significant group differences. Scores improved from baseline to postintervention and follow-up (both *P*<.001), with no change between postintervention and follow-up (*P*=.115).

**Figure 1. Trajectory of Depression and Anxiety Scores Across Time by Intervention Group**

**Figure 2. Trajectory of Affects Balance Across Time by Intervention Group**

**Figure 3. Trajectory of Interpersonal Relationship Across Time by Intervention Group**

**Figure 4. Trajectory of Cognitive Emotional Regulation- Adaptive Strategy Across Time by Intervention Group**

**Figure 5. Trajectory of Cognitive Emotional Regulation- Maladaptive Strategy Across Time by Intervention Group**

**Figure 6. Trajectory of Mindfulness Across Time by Intervention Group**


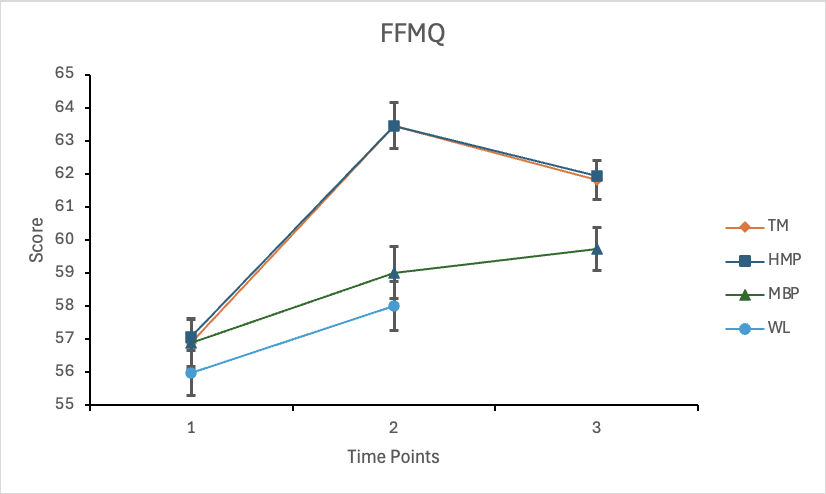


***Note*. The line graphs display changes in mean scores for each psychological outcome at three time points: baseline (T1), post-intervention (21 days, T2), and 3-month follow-up (T3), across the four intervention groups: Traditional Mindfulness (TM), Habitual Mindfulness Practice (HMP), Mindfulness-Based Psychoeducation (MBP), and Waitlist Control (WL). Error bars represent standard errors of the mean.**

Across outcomes, depressive and anxiety symptoms decreased markedly from T1 to T2 in all intervention groups, with reductions maintained or slightly further improved at T3, whereas the WL group showed relatively stable or higher symptom trajectories over time. TM and HMP consistently demonstrated larger and more sustained reductions in symptoms compared with MBP and WL.

In contrast, mindfulness levels and adaptive emotion regulation increased from T1 to T2 and were largely maintained at T3, particularly in TM and HMP. Maladaptive emotion regulation and interpersonal difficulties decreased over time across all intervention conditions, with more pronounced improvements in active intervention groups compared with WL.

## References

No references cited.
